# Comparison of Prostate-Specific Antigen and Its Density and Prostate Health Index and Its Density for Detection of Prostate Cancer

**DOI:** 10.3390/biomedicines11071912

**Published:** 2023-07-06

**Authors:** Youngjun Boo, Jae Hoon Chung, Minyong Kang, Hyun Hwan Sung, Hwang Gyun Jeon, Byong Chang Jeong, Seong Il Seo, Seong Soo Jeon, Hyun Moo Lee, Wan Song

**Affiliations:** Department of Urology, Samsung Medical Center, Sungkyunkwan University School of Medicine, Seoul 06351, Republic of Korea; youngjun91.boo@samsung.com (Y.B.);

**Keywords:** prostate cancer, prostate-specific antigen, prostate-specific antigen density, prostate health index, prostate health index density

## Abstract

As the incidence of prostate cancer (PCa) has increased, screening based on prostate-specific antigen (PSA) has become controversial due to the low specificity of PSA. Therefore, we investigated the diagnostic performance of prostate health index (PHI) density (PHID) for the detection of PCa and clinically significant PCa (csPCa) compared to PSA, PSA density (PSAD), and PHI as a triaging test. We retrospectively reviewed 306 men who underwent prostate biopsy for PSA levels of 2.5 to 10 ng/mL between January 2020 and April 2023. Of all cohorts, 86 (28.1%) and 48 (15.7%) men were diagnosed with PCa and csPCa, respectively. In ROC analysis, the highest AUC was identified for PHID (0.812), followed by PHI (0.791), PSAD (0.650), and PSA (0.571) for PCa. A similar trend was observed for csPCa: PHID (AUC 0.826), PHI (AUC 0.796), PSAD (AUC 0.671), and PSA (0.552). When the biopsy was restricted to men with a PHID ≥ 0.56, 26.5% of unnecessary biopsies could be avoided; however, 9.3% of PCa cases and one csPCa case (2.1%) remained undiagnosed. At approximately 90% sensitivity for csPCa, at the given cut-off values of PHI ≥ 36.4, and PHID ≥ 0.91, 48.7% and 49.3% of unnecessary biopsies could be avoided. In conclusion, PHID had a small advantage over PHI, about 3.6%, for the reduction in unnecessary biopsies for PCa. The PHID and PHI showed almost the same diagnostic performance for csPCa detection. PHID can be used as a triaging test in a clinical setting to pre-select the risk of PCa and csPCa.

## 1. Introduction

As the incidence of prostate cancer (PCa) has increased [1], PCa screening based on prostate-specific antigen (PSA) has become controversial [2,3]. Owing to the low specificity of PSA for PCa detection [4], there are concerns regarding the over-diagnosis and over-treatment of PCa, and their impact on the lifetime of patients [5,6]. In addition, unnecessary prostate biopsies were performed for men with suspected PCa. Therefore, many efforts have been made to identify new biomarkers that could improve PCa detection. PSA density (PSAD) is a clinically useful tool to compensate for the low sensitivity of PSA, and recent studies have reported that PSAD is effective in determining whether to perform a prostate biopsy and improves the detection of PCa and clinically significant PCa (csPCa) [7,8,9].

In the past decade, the prostate health index (PHI) was introduced, and the Food and Drug Administration (FDA) approved the PHI for PCa in 2012 [10]. PHI is calculated using the total PSA (tPSA), free PSA (fPSA), and [−2] pro-PSA (p2PSA), and it outperforms tPSA or fPSA for the detection of PCa and csPCa [11,12]. In addition, it has been reported that PHI is significantly correlated with tumor volume and prognosis, such as tumor recurrence after radical prostatectomy [13,14]. Furthermore, similar to the concept of PSAD, PHI density (PHID) was introduced in 2014 [15]. The outcomes of recent studies showed that PHID outperformed PHI for the detection of csPCa in the area under the receiver operating characteristic (ROC) curve (AUC) analysis (AUC 0.84 vs. AUC 0.76) [16], and could predict aggressive pathological results after radical prostatectomy [17].

Recently, several studies have reported the clinical utility of PHI and PHID for the detection of PCa and csPCa. However, the results of these studies have been inconsistent. One prospective study demonstrated that PHID has an advantage in the detection of PCa compared with PHI (AUC 0.835 vs. 0.801, *p* = 0.0013) but showed equal performance for csPCa [18]. On the other hand, there were no differences in detection of PCa between PHID and PHI regarding the AUC (0.77 vs. 0.79 and 0.737 vs. 0.749) [19,20]. Therefore, it is necessary to investigate the value of PHID in comparison to PHI for the detection of PCa and csPCa.

Approximately 1–4% of men experience infectious complications after prostate biopsy, and a much larger number of patients suffer post-biopsy complications, including minor complications, such as hematuria and voiding difficulty [21]. However, early diagnosis and active treatment of csPCa are also important because of its potentially lethal tendencies. The above-mentioned diagnostic biomarkers can help to avoid unnecessary prostate biopsies and aid in the diagnosis of csPCa, especially in men with serum PSA levels between 2.5 and 10 ng/mL, the so-called “gray zone”. Therefore, in this study, we evaluated the diagnostic performance of PHID in the detection of PCa and csPCa and compared it with that of PSA, PSAD and PHI as triaging tests.

## 2. Materials and Methods

### 2.1. Study Design

This study was approved by the Institutional Review Board of the Samsung Medical Center (IRB No. 2023-03-049). Informed consent was waived owing to the retrospective study design, and we provided an opportunity to opt-out for men who did not wish to provide personal information. All study protocols complied with the Declaration of Helsinki.

We retrospectively evaluated 327 men who underwent prostate biopsy for suspected PCa between January 2020 and April 2023. All the men were naïve to a previous prostate biopsy. The inclusion criteria were as follows: (1) men with serum PSA levels between 2.5 and 10 ng/mL or abnormal digital rectal examination (DRE), (2) who underwent transrectal ultrasound (TRUS)-guided prostate biopsy of at least systemic 12 cores and additional cognitive-targeted biopsy was performed if a hypoechoic lesion was identified. The exclusion criteria were as follows: (1) men who had previously undergone prostate surgery such as transurethral resection of the prostate or Holmium laser enucleation of the prostate; (2) men with urinary tract infection or acute bacterial prostatitis; and (3) men who were treated with 5-alpha reductase inhibitors. None of the cohorts underwent multiparametric magnetic resonance imaging (mpMRI) before TRUS-guided prostate biopsy. Of the entire cohort, 21 men were excluded because of incomplete clinical data, and 306 men were included in the final analysis.

### 2.2. Laboratory Tests

As a prostate biopsy may lead to PSA elevation, all blood samples were collected before the prostate biopsy. Serum samples were stored at −80 °C, centrifuged at 1500× *g* for 15 min within three hours of sampling, and stored at −20 °C until analysis. A Beckman Coulter DxI 800 Immunoassay System was used to analyze the tPSA, fPSA, and p2PSA levels. The PHI was calculated according to the following formula: PHI = (p2PSA/fPSA) × (tPSA^1/2^) [22]. The prostate volume was estimated via TRUS by applying the ellipsoid formula: width × length × height × 0.52. PSAD and PHID were calculated with (tPSA/prostate volume) and (PHI/prostate volume), respectively.

### 2.3. Biopsy Protocol and Histopathological Analysis

Before the biopsy, a rectal preparation (bisacodyl suppository) was performed. All biopsies were performed as outpatient procedures under local anesthesia. For prophylactic antibiotics, oral quinolone and/or 3rd cephalosporin was administered for 7 days from the day before the procedure. A cognitive-targeted biopsy of more than one core sample was performed for suspicious lesions initially; then, a 12-core systemic biopsy was performed with an 18-gauge core biopsy needle mounted on an automatic biopsy gun in a two-dimensional plane (axial and sagittal) TRUS probe (BK Medical, Herlev, DenmarkTransducer 8818).

The biopsy specimens were reviewed by a pathologist with 15 years of experience specializing in uro-oncology and reported according to the guidelines of the 2014 International Society of Urological Pathology (ISUP) Consensus Conference [23]. Gleason grade (GG) 1 is equivalent to a Gleason score (GS) of 3 + 3 = 6, GG 2 is equivalent to GS 3 + 4 = 7, GG 3 is equivalent to GS 4 + 3 = 7, GG 4 is equivalent to GS 4 + 4 = 8, and GG 5 is equivalent to GS 9–10. In our study, csPCa was defined as GG ≥ 2.

### 2.4. Statistical Analysis

Continuous variables were reported as median (interquartile range [IQR]) and mean (standard deviation [SD]). Statistical differences were assessed using an independent Student’s *t*-test. Univariate and multivariate logistic regression analysis was used to determine the association between measured variables and PCa and csPCa. Odds ratios (OR) with 95% confidence intervals (CI) were also calculated. The diagnostic performance of the evaluated variables was quantified using the area under the ROC curve, and the DeLong test was used to examine the differences between AUCs. The sensitivity, specificity, and positive and negative predictive values (PPV and NPV) of the evaluated variables were calculated at different cut-off values for the detection of PCa and csPCa. Statistical analyses were performed using IBM SPSS Statistics (version 23.0; IBM Corp., Armonk, NY, USA) and MedCalc version 14.8.1 (MedCalc Software, Ostend, Belgium). Statistical significance was set at a two-sided *p*-value < 0.05.

## 3. Results

### 3.1. Baseline Demographic and Clinicopathologic Characteristics

The baseline characteristics of all cohorts are provided in Table 1. Of all the cohorts, the median (IQR) age was 65.0 (60.0–70.0) years, and the PSA was 5.66 (4.03–7.23) ng/mL. The median prostate volume and PHI were 37.9 (28.3–53.5) mL and 38.0 (28.6–49.4), respectively. Of the 306 men included, PCa was identified in 86 (28.1%), and 48 out of 306 (15.7%) were diagnosed with csPCa. When all patients were divided into two groups according to PCa detection (no vs. yes), the prostate volume (41.4 mL vs. 30.6 mL, *p* < 0.001) was smaller in men with PCa than that in the non-PCa group. As regards biomarkers, the total PSA level was similar between the two groups (5.79 ng/mL vs. 5.27 ng/mL, *p* = 0.362); however, men with PCa had a significantly higher PSAD (0.14 vs. 0.17), PHI (33.5 vs. 51.4) and PHID (0.81 vs. 1.68) (all *p* < 0.001).

### 3.2. Diagnostic Ability of Each Biomarker

Univariate logistic regression analysis for the prediction of PCa revealed that age (OR 1.049, *p* = 0.031), abnormal DRE (OR 1.910, *p* < 0.001), and PHI (OR 3.216, *p* < 0.001) were significant predictors of PCa. When prostate volume was combined with biomarkers, PSAD and PHID were also significant predictors of PCa (OR 1.072, *p* < 0.001 and OR 3.861, *p* < 0.001). In addition, multivariate analysis adjusted for age and abnormal DRE revealed that the PSA level did not predict PCa (*p* = 0.317), but PSAD, PHI, and PHID were clinically significant predictors of PCa (all *p* < 0.001) (Table 2). When analyzed for csPCa, age (OR 1.042, *p* = 0.176) and PSA level (OR 0.952, *p* = 0.512) were not significant predictors, but abnormal DRE (OR 1.651, *p* < 0.001) and PHI (OR 2.423, *p* < 0.001) were significantly associated with the prediction of csPCa. PSAD and PHID, which are biomarkers considering prostate volume, were also clinically significant predictors of csPCa (all *p* < 0.001). Similar to the results in PCa, when adjusted for age and abnormal DRE, PSAD, PHI, and PHID were clinically significant predictors of csPCa (all *p* < 0.001), but not PSA (*p* = 0.523) (Table 3). The percentage accuracy in classification was 72.2% for PCa and 84.5% for csPCa, respectively.

**Figure 1:**
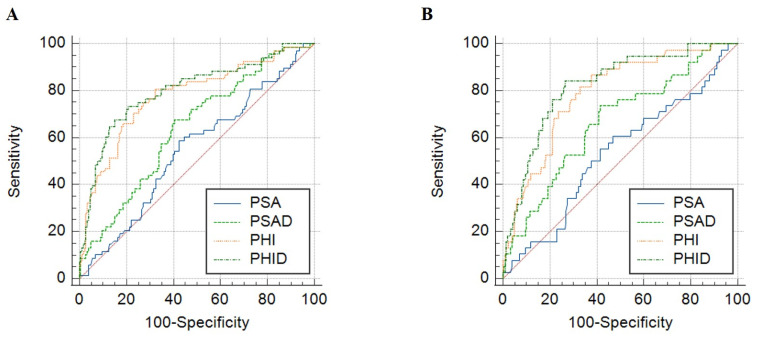
Receiver operating characteristic (ROC) curve analysis for predicting (**A**) overall prostate cancer and (**B**) clinically significant prostate cancer. Red line is reference.

When applying ROC analysis to examine the ability of each diagnostic biomarker to detect PCa, the highest AUC was identified for PHID (AUC 0.812), followed by PHI (AUC 0.791), PSAD (AUC 0.650), and PSA (AUC 0.571) (Figure 1A). The AUC for PHID and PHI were significantly larger than those for PSAD (all *p* < 0.001) and PSA (all *p* < 0.001). However, AUC difference between PHID and PHI was not significant (*p* = 0.221). When csPCa was analyzed as an outcome, a similar order was observed: PHID (AUC 0.826), PHI (AUC 0.796), PSAD (AUC 0.671), and PSA (0.552) (Figure 1B). Similar to the results in PCa, the AUC of PHID and PHI was significantly larger than that of PSAD (*p* < 0.001 and *p* = 0.0021, respectively) and PSA (all *p* < 0.001). However, the AUC between PHID and PHI was not significantly different (*p* = 0.191).

### 3.3. Diagnostic Performance According to Different Cut-Off

Table 4 summarizes the diagnostic performance of each biomarker for detecting PCa and csPCa. When applying the biomarker cut-offs that allowed for approximately 90% diagnostic sensitivity for PCa, the diagnostic specificity of PHID (32.7%) was higher than that of PHI (30.0%), PSAD (22.7%), and PSA (6.8%). When the biopsy was restricted to men with PHID ≥ 0.56, 26.5% of unnecessary biopsies could be avoided; however, 9.3% of PCa cases and one csPCa case (2.1%) remained undiagnosed.

Furthermore, when biomarker cut-offs that allowed for approximately 90% diagnostic sensitivity for csPCa were applied, PHI (55.4%) and PHID (56.2%) showed higher diagnostic specificity for csPCa than that shown by PSA (910.1%) or PSAD (20.9%). At cut-off values of PSA ≥ 3.26 ng/mL, PSAD ≥ 0.090, PHI ≥ 36.4, and PHID ≥ 0.91, 10.1%, 19.6%, 48.7%, and 49.3% of unnecessary biopsies could be avoided. There was little difference in the number of avoidable biopsies between PHI (*n* =149) and PHID (*n* = 151), and the number of undiagnosed PCa remained the same (*n* = 14).

Table 5 presents the percentages of PCa and csPCa according to the different PHID cut-off values. We identified a positive correlation between the PHID cut-off and PCa or csPCa ratio. If the value of PHID was 1.2–1.5, approximately one-third of men had a probability of PCa, and the risk of csPCa was 23.3%. For PHID values of >1.5, the probability of PCa and csPCa increased to two-thirds and approximately 40% of men, respectively.

## 4. Discussion

In this study, we analyzed the diagnostic performance of PSA, PSAD, PHI, and PHID in the detection of PCa and csPCa. Of the 306 men included, 86 (28.1%) and 48 (15.7%) patients were diagnosed with PCa and csPCa, respectively. We found that PHID showed the highest diagnostic performance for PCa and csPCa, and could reduce the number of unnecessary biopsies. In addition, when we categorized PHID according to different cut-off values, a positive correlation between the PHID cut-off and PCa or csPCa ratio was identified. Therefore, it could be used as a triaging test in a clinical setting to pre-select the risks of PCa and csPCa.

In clinical practice, mpMRI is an essential tool, and its usefulness is gradually increasing in the diagnosis of PCa [24,25]. Park et al. reported that the interpretation of mpMRI using prostate imaging reporting and data system version 2 (PI-RADSv2) is useful for the stratification of the risk of PCa and csPCa [26]. The detection rates of PCa and csPCa were 63% and 46% in PI-RADSv2 category 4, and 90% and 75% in PI-RADSv2 category 5, respectively. However, about 10–20% of csPCa are not visible in PI-RADSv2 category 1–2 [27]. In addition, mpMRI is not cost-effective and interobserver disagreements should be considered in the interpretation of mpMRI [28]. Therefore, a biomarker is required that could be used as a triaging test to reduce the number of unnecessary tests while supplementing the PSA shortcomings.

The PHI was developed to compensate for the limitations of PSA and was approved by the FDA for PCa in 2012. Le et al. reported the first study on the clinical utility of PHI in a prospective PCa screening study [29], in which PHI had the best overall performance (AUC: 0.770) to discriminate PCa from benign disease in men with PSA levels between 2.5 and 10 ng/mL and a negative DRE. In addition, Agnello et al. reported that PHI showed high accuracy in PCa detection and was a very useful biomarker that could distinguish the aggressiveness of PCa [30]. However, prostate volume is an important factor in the interpretation of biomarkers used for the detection of PCa. Filella et al. reported that the AUC of PHI for PCa detection was 0.818 when the prostate volume was less than 35 cc, while it was 0.716 and 0.654 when the prostate volume was 36–50 cc and more than 50 cc, respectively [31].

In 2014, similar to the concept of PSAD, PHID was introduced [15]. Tosoian et al. explored the utility of PHID in 118 men who underwent prostate biopsy and reported that PHID had the highest differential ability for csPCa (AUC 0.84), thus avoiding 38% of unnecessary biopsies and missing only 2% of csPCa [16]. However, several points must be considered. First, PHI and PHID demonstrated discrepancies in the detection of PCa and csPCa. Stephan et al. reported that PHID has an advantage in the detection of PCa compared to PHI (AUC 0.835 vs. 0.801; *p* = 0.013) [18]. In contrast, Friedl et al. [19] and Peters et al. [20] showed no differences in the detection of PCa regarding the AUC (0.77 vs. 0.79 and 0.737 vs. 0.749) between PHID and PHI, respectively. In our study, PHID showed the highest AUC for PCa (AUC 0.812) and csPCa (AUC 0.826), but did not significantly differ from that of PHI (AUC 0.791, *p* = 0.221 and AUC 0.796, *p* = 0.191, respectively)

Furthermore, a consensus on the optimal PHID cut-off value is not available. Garrido et al. reported that when applying the biomarker cut-off that allowed for approximately 90% of diagnostic sensitivity for csPCa, 26.3% of unnecessary biopsies could be avoided at PHID ≥ 0.49 [32]. In a study by Chiu et al., at 90% sensitivity, 43.7% of unnecessary biopsies could be avoided when the PHID was >0.67 [33]. In our study, with a cut-off of PHID ≥ 0.91, PHID had a 56.2% specificity, and 49.3% of unnecessary biopsies could be avoided at the cost of 8.3% of csPCa. Therefore, we present a comprehensive table to describe the percentage of PCa and csPCa at different PHID cut-off ranges to compensate for standards that are not clearly defined. Our study showed that for PHID of 1.2–1.5, approximately one-third of men had a probability of PCa, with a 23.3% risk of csPCa. For PHID values of >1.5, the probability of PCa and csPCa increased to two-thirds and approximately 40% of patients, respectively.

In this study, when applying the biomarker cut-offs that allowed for approximately 90% of diagnostic sensitivity, the NPV of PHI and PHID was 89.2% and 90.0% for PCa, and 97.3% and 97.3% for csPCa, respectively. However, the PPV was relatively low at approximately 33.6–34.5% for PCa and 27.7–28.0% for csPCa, because it can be changed depending on the situation, such as the incidence of PCa. In our study, PCa and csPCa were identified in 28.1% (86/306) and 15.7% (48/306) of men, respectively, with serum PSA levels between 2.5 and 10 ng/mL. These real descriptors of the diagnostic test performance are expected to improve through machine learning in the future.

This study had several limitations. First, an inherent selection bias may exist because of the retrospective design of this study, which was performed at a single institution. However, this study examined a prospectively collected database that reflected real-world clinical practices. Second, the sample size of the cohort was relatively limited; therefore, the diagnostic performance of each variable requires further validation with a larger cohort to corroborate the findings reported in the present study. Third, in our study, the prostate volume was estimated via TRUS using an ellipsoid formula. However, since some differences may occur depending on the method of measuring prostate volume, it should be taken into account when interpreting the diagnostic performance of PHID. Finally, we examined the outcomes of a cognitive-targeted biopsy combined with a 12-core systemic biopsy performed by an expert urologist; however, applying other biopsy methods might yield different results. Therefore, confirmation via a large prospective study is required to verify the results reported herein.

## 5. Conclusions

In men with a PSA level between 2.5 and 10 ng/mL, PHI and PHID showed higher diagnostic performance for PCa and csPCa than PSA and PSAD. For the detection of PCa, the diagnostic performance of PHID was not significantly different from that of PHI. However, PHID had a small advantage over PHI, about 3.6%, for the reduction in the number of unnecessary biopsies. The PHID and PHI showed almost the same diagnostic performance for csPCa detection. We identified a positive correlation between the PHID cut-off and PCa or csPCa ratio. Therefore, it can be used as a triaging test in a clinical setting to pre-select the risk of PCa and csPCa and reduce the number of unnecessary tests while supplementing the shortcomings of PSA. Based on this study, further studies should be conducted to provide evidence that can more clearly predict the risk of PCa and csPCa through a combination of serum biomarkers and the result of mpMRI.

## Figures and Tables

**Table 1:** Baseline characteristics of patients.

Variables	Total	Detection of Prostate Cancer		*p*
		No	Yes	
No. of patients, n (%)	306	220 (71.9)	86 (28.1)	
Age, years				0.031
Median (IQR)	65.0 (60.0–70.0)	64.0 (59.0–69.0)	66.0 (61.0–72.0)	
Mean (SD)	64.5 (8.1)	63.9 (8.3)	66.1 (7.2)	
Abnormal DRE, n (%)				<0.001
No	245 (80.1)	194 (88.2)	51 (59.3)	
Yes	61 (19.3)	26 (11.8)	35 (40.7)	
Total PSA, ng/mL				0.362
Median (IQR)	5.66 (4.03–7.23)	5.79 (4.03–7.36)	5.27 (3.93–7.26)	
Mean (SD)	5.81 (1.99)	5.88 (2.04)	5.64 (1.94)	
Prostate volume, mL				<0.001
Median (IQR)	37.9 (28.3–53.5)	41.4 (32.2–56.2)	30.6 (23.4–39.6)	
Mean (SD)	43.6 (21.6)	47.3 (22.1)	35. 1(15.0)	
PSAD				<0.001
Median (IQR)	0.15 (0.10–0.20)	0.14 (0.09–0.18)	0.17 (0.13–0.21)	
Mean (SD)	0.15 (0.08)	0.13 (0.07)	0.18 (0.09)	
PHI				<0.001
Median (IQR)	38.0 (28.6–49.4)	33.5 (25.9–42.7)	51.4 (40.5–70.9)	
Mean (SD)	42.0 (21.4)	36.4 (16.2)	59.2 (26.0)	
PHID				<0.001
Median (IQR)	1.00 (0.59–1.60)	0.81 (0.53–1.15)	1.68 (1.07–2.58)	
Mean (SD)	1.21 (0.98)	0.97 (0.64)	1.98 (1.25)	
Gleason Grade, n (%)				
GG 1			38 (44.2)	
GG 2			25 (29.1)	
GG 3			6 (7.0)	
GG 4			9 (10.4)	
GG 5			8 (9.3)	

**Table 2:** Multivariate logistic regression analysis for prediction of PCa.

Parameters	Multivariate Analysis Adjusted for Age and Abnormal DRE		
	OR	95% CI	*p*
PSA	0.927	0.815–1.153	0.317
PSAD	1.523	1.261–1.875	<0.001
PHI	4.261	3.239–7.121	<0.001
PHID	4.951	3.550–7.920	<0.001

**Table 3:** Multivariate logistic regression analysis for prediction of csPCa.

Parameters	Multivariate Analysis Adjusted for Age and Abnormal DRE		
	OR	95% CI	*p*
PSA	0.962	0.854–1.321	0.523
PSAD	1.642	1.462–1.906	<0.001
PHI	2.871	1.926–3.926	<0.001
PHID	3.419	2.503–4.762	<0.001

**Table 4:** Diagnostic performance of prostate-specific antigen, prostate-specific antigen density, prostate health index, and prostate health index density for the detection of prostate cancer and clinically significant prostate cancer.

**Prostate Cancer**								
Predictor	Cut-off	Sensitivity (95% CI)	Specificity (95% CI)	PPV (95% CI)	NPV (95% CI)	Biopsy avoided, n (%)	PCa missed, n (%)	csPCa missed, n (%)
PSA	≥3.20	90.7% (82.5–95.9)	6.8% (3.9–11.0)	27.6% (22.4–33.2)	65.2% (42.7–83.6)	23 (7.5)	8 (9.3)	3 (6.3)
PSAD	≥0.088	90.7% (82.5–95.9)	22.7% (17.4–28.8)	31.5% (25.7–37.6)	86.2% (74.6–93.9)	59 (19.3)	8 (9.3)	3 (6.3)
PHI	≥28.5	90.7% (82.5–95.9)	30.0% (24.0–36.5)	33.6% (27.6–40.1)	89.2% (79.8–95.2)	70 (22.9)	8 (9.3)	2 (4.2)
PHID	≥0.56	90.7% (82.5–95.9)	32.7% (26.6–39.4)	34.5% (28.3–41.1)	90.0% (81.2–95.6)	81 (26.5)	8 (9.3)	1 (2.1)
**Clinically significant prostate cancer**								
Predictor	Cut-off	Sensitivity (95% CI)	Specificity (95% CI)	PPV (95% CI)	NPV (95% CI)	Biopsy avoided, n (%)	PCa missed, n (%)	csPCa missed, n (%)
PSA	≥3.26	91.7% (80.0–97.7)	10.1% (6.7–14.4)	15.9% (11.8–20.8)	86.7% (69.3–96.2)	31 (10.1)	9 (10.5)	4 (8.3)
PSAD	≥0.090	91.7% (80.0–97.7)	20.9% (16.1–26.4)	17.7% (13.2–23.1)	93.1% (83.3–98.1)	60 (19.6)	9 (10.5)	4 (8.3)
PHI	≥36.4	91.7% (80.0–97.7)	55.4% (49.1–61.6)	27.7% (20.9–35.3)	97.3% (93.2–99.3)	149 (48.7)	14 (16.3)	4 (8.3)
PHID	≥0.91	91.7% (80.0–97.7)	56.2% (49.9–62.4)	28.0% (21.2–35.7)	97.3% (93.3–99.3)	151 (49.3)	14 (16.3)	4 (8.3)

**Table 5:** Percentage of prostate cancer and clinically significant prostate cancer diagnosed at different cut-off values of prostate health index density.

	PHID Cut-Off				Total
	<0.9	0.9–1.2	1.2–1.5	>1.5	
PCa	9.7% (15/154)	20.5% (9/44)	33.3% (10/30)	66.7% (52/78)	28.1% (86/306)
csPCa	3.9% (6/154)	9.1% (4/44)	23.3% (7/30)	39.7% (31/78)	15.7% (48/306)

## Data Availability

The dataset used and/or analyzed during the current study is available from the corresponding author upon reasonable request.
